# Hay Fever Discussion

**Published:** 1887-09

**Authors:** 


DISCUSSION BY MEMBERS OF THE I. H. A.

Dr. Nash—I want to say of this paper on hay fever that I was particularly interested in it, as I have been frequently afflicted with hay fever for thirty years of my life. It has been said that some cases of hay fever were curable with Lycopodium. The cases cured in that way would not stay cured the next year. I have been a victim of this disease, and have been several times relieved, and never had it return until the next year at the same time, which was between the twentieth and twenty-third of August. When I was relieved, and when I had found the homœopathic remedy that completely covered my case in any particular year and took it, then I was relieved for the season, completely relieved, quickly relieved.

Lachesis cured me one year, and cured me right in the midst of a ride in the country, which is the worst exercise I can engage in when I am afflicted. The indications were simply that when I laid down and took a nap, day or night, my complaint was fearfully aggravated every time when I woke up. That year Lachesis cured me, and cured me within three hours, so that I was completely cured with all the symptoms connected with it that I was subjected to. A year later on I was cured again. I was cured and have remained comparatively free of the June attacks which used to afflict me in those days by Gelsiminum. The attack always came on in the morning with a tingling and watery discharge, which is a characteristic for the remedy. Now I have not had that condition since that time, except in a very slight degree, and then Gelsiminum relieved me. The following year I was relieved by Carbo veg., and made use of it. The symptom was pain and extreme tingling in the nose. I did suffer from it fearfully. And I am so afraid that I will not get my remedy that I go to the mountains every year to get rid of it, where I am perfectly free from it. I was thinking, when hearing the case which was relieved on the 27th day of September, "Now if during the course of this disease a heavy frost comes on, enough to kill all the corn in the country round about, I am relieved in twenty-four hours." I think sometimes that that had something to do with it.

Dr. Biegler—I would state that that is an important point, and that I took particular pains to watch for that very occurrence, and also the gentleman, who is a very intelligent man, a professor and teacher in the Institute ; he also keeps a record of the weather, and there was no frost at the time. He is well aware of that. He knows that, and he knows that he gets immediate relief as soon as a severe frost occurs, therefore he always kept watch of it; and he has been very much interested in the new homœopathic treatment of this disease, as he has never had it before; therefore he has kept watch of all these circumstances, and the relief came within twenty-four hours—the next day he was relieved, not only of this intolerable itching but altogether, and he has been marveling ever since at the relief that he obtained.

Dr. Nash—In one year I was completely relieved by Sticta pul. The pain then was immediately in this region—the frontal region. I generally have no stoppage whatever, but the discharge is profuse. In this case it stopped when I was relieved by this medicine. When I find an attack coming on I study it carefully and give the right remedy.

Dr. Wesselhœft—I want to have a word to say about the nature of this disease. I do not want to be personal, but Dr. Nash has got a chronically red nose.

Dr. Nash—I always have had it.

Dr. Wesselhœft—I also have a patient who has a chronically red nose, and he also has the hay fever. As soon as the hay fever comes on the red nose recedes until the hay fever has passed away.

Dr. Nash—Mine don't. My nose grows redder.

Dr. Wesselhœft—I believed this, and I believe it yet. I know that this "hay cold" is nothing but a suppression of psora at a certain time of the year, brought on, as we know is the case, with certain forms of salt rheum, which are brought on at certain times of the year. I think that this is a salt rheum form or psoric form of a disease in the nasal passages, and for this reason I will explain my position—we have treated a good many cases with more or less good results. The cases have been relieved and one cured, but in each case eczema appeared on the skin—in one case on the forehead, and in the other on the scrotum. Now we have a right to suppose that at certain times of the year, under certain circumstances, this psoric tendency in certain individuals is brought on in the Schneiderian membrane and the membrane of the throat and lungs by certain conditions which we do not know about, and that instead of its coming out to the surface of the dermis it comes only as far as the internal skin and the mucous membrane. To make my position still more clear I wish to remark this, that I had one patient who always had eczema come out on the wrist with the first snow flurry of the autumn. So sure was she of this that she dreaded the first snow storm, for the salt rheum would start back of the wrist and extend to the hand with the most intense itching. I never have had any occasion to observe that personally when the first snow storm was the cause of it; but on one occasion I was called to see this eczema, and she said to me, "Doctor, this is the first time in my life that I have had this eczema without a snow storm." Said I, "That breaks up the whole theory, and it does not make much matter whether it does or not." After I went home, almost immediately I met my milkman at the door, and he said to me, "Good-morning, Doctor, we had a nice little snow flurry this morning. Did you know it?" [Laughter.] Well, I could not help getting into my carriage again and driving back to the house to tell the lady that the snow flurry was the thing, after all, and that it had occurred at three o'clock.

Now these are conditions that we cannot explain, because we do not know, but I never personally have ever taken any stock in this idea of the pollen and the material influence producing this condition. I think that they are periodical attacks excited by perhaps certain conditions of the atmosphere, but they are in the person, and they do not go flying outside and poison us. [Applause.]

Dr. Nash—That is what I believe myself; but in spite of the Doctor's theory about psoric nature of the disease, I would state, in addition, that when I have this disease my mouth and throat, and even down into my lungs, if you could see there, they have the same sensation as if broken out with a rash as thick as you will find in the measles. As far as any disease on the skin is concerned I have never had anything like eczema that I know of.

Dr. Bell—Did you have the itch?

Dr. Nash—I suppose I did, but I did not know about it. My mother never told me.

Dr. Bell—I guess you did. I had it.

Dr. Nash—I will never deny it, anyway. Now, there is one more thing in regard to corn being the cause of it. I don't believe that it is the cause of it, the primary cause—I mean it is the *exciting* cause. The reason for that is, that when I am on the mountains thirty miles from any corn—when I am up in the Blue Mountains—I have no more manifestations of it.

Dr. Bell—It is very troublesome and difficult, that is all.

Dr. Allen—I would like to add to what Dr. Wesselhœft has said that I think he has the key to it largely. This is a psoric difficulty. The trouble is in the patient. In order to remove the disease you must treat the patient before the time of the attack, and so render the system impervious to the external influences which predispose him to the attack. I have treated a large number of cases of hay fever. The annual hegira from my town a few years ago was wonderfully large. A large number of the people now stay at home and attend to their duties, domestic or otherwise, and they are not bothered at all at the time of their annual attack of hay fever. I insist on their coming to see me two or three months before the time of the attack coming on. I prepare them—I prepare the system—to resist this on-coming attack, and they go through it without the usual occurrence of the disease. But what has disturbed me in the last few years is a few cases which are aggravated by the hot weather. For instance, when the hot days, particularly of May, come along, on comes an attack of hay fever if they are out and exposed; it is necessary for them to stay in the house, and then they are not subject to it at all. I have two now under treatment. For weeks they never think of going down town, except after sundown or on a cloudy day. They cannot bear exposure to the sun at all; it will bring on a most violent attack of coryza, a sensation of itching in their nasal and lachrymal passages. I have found Natrum-muriaticum the best remedy for this affection, or the remedy most frequently indicated; but I do not like that phrase, as there is no such thing as the best remedy. It is the remedy where the exposure to the heat of the sun will bring it on, or exposure to the heat of a cooking-stove, especially with ladies who are compelled to do their own cooking. Dr. Nash needs a dose or two of Psorinum. He will be cured if he will take that.

Dr. Nash—At this time of the year?

Dr. Allen—Not only at this time of the year, but at other times of the year when this begins to come on these people cannot go out in hot weather. It is the exciting cause, just, for instance, when a lady is in the right condition of system she may be attacked with any other disease.

Dr. Brown—What potency will you give of the Psorinum?

Dr. Allen—My favorite potency for years, and the potency I first gave when I obtained it, is the 42M. It has stood by me and I have by it. Lately I have used one of the CM potencies. That sometimes relieves. Whenever that apparently loses its hold I give a few doses of Psorinum, and then of something else I think will be of service, possibly a dose or two of Sulphur. I may give Psorinum or something antipsoric. I am bound to give the indicated remedy. I give the indicated remedy as near as I can find it from the totality of the symptoms. I thus prepare my patient so that the system is not affected by the attack of this morbific matter. This is not only true in respect to hay fever, but it is true of almost all other difficulties—scarlet fever, measles, etc.—and a comparatively healthy person is impervious to the attacks of these influences—is capable of throwing them off. And Hahnemann has given us antipsorics in his antipsoric treatment in his *Chronic Diseases*, and by that means, if we properly understand it and thoroughly know what to do, we can eradicate the majority of these difficulties. I have been told in reference to this work on chronic diseases that it was a fact that a great many physicians did not have a copy of the *Chronic Diseases* at all, and they know nothing about Hahnemann's treatment of chronic diseases. Hence, the annual hegira of our hay fever patients. Some hay fever patients wake up in the morning and have an attack. For instance, one lady has an attack as soon as the mountain ash blooms—it is one of our flowering shrubs—and she will wake up and have an attack. When the mountain ash is in bloom on comes the attack of hay fever. She is sure to have it. In another case another patient would have it from the Western rag weed, which is the producing cause. Another from the clover, the melilotus; another from the cornfield, and another from new-cut hay, and another from something else, and so forth and so on, but the difficulty is with the attack of hay fever and not the patient. Consequently we lose somewhat of what might be our most brilliant triumph.

Dr. Biegler—When this article is published it will be noticed that the prescription is decidedly based upon the indicated remedy, and this was understood, so it cannot appear as a remedy for hay fever, which was not the intention. The intention was to mark that agent or symptom which is given in this proving.

Dr. Allen—I did not intend to reflect upon that.

Dr. Biegler—I understand that, and I will add that this year I have taken that advice and suggested to a gentleman who came to me some two or three weeks ago for treatment, not having the disease, and this time I made out Colchicum. What the result may be I do not know. In regard to fashionable traveling and people going to certain places, it reminds me of a season that I spent in this beautiful region here along the Jersey coast at Beach Haven. When I arrived there I found a lot of people sneezing. They were sneezing on the piazza, they sneezed in the dining-room, and they sneezed everywhere, although that was said to be the haven for hay fever cases. I was there a week, and those who were there would say good-morning to me sneezing, and good-night to me sneezing, and I remained there three weeks and it was a good-night sneezing and a good-morning sneezing, and yet they were happy, sneezing all the time. [Laughter.]

Dr. Hitchcock—I would like to corroborate what Dr. Wesselhœft has said in regard to hay fever being a psoric condition. I have been a sufferer considerably from hay fever for a good many years. When I was a schoolboy I had an eruption on my thumb, regular salt rheum, which lasted for years, and finally that disappeared. When I went to college I there contracted a very severe cold in the head, coryza, and from that time I have suffered from hay fever every year. But there is this peculiar alternation of symptoms; during the time when I am not affected by the hay fever over the lower end of the sacrum there is a scaly eruption, but when the hay fever comes on that disappears and the skin is perfectly smooth. Last summer at Saratoga at the meeting of the Association I had a severe attack of pain in the maxillary joint on the right side. I asked Dr. Ballard and two or three others about it, and also told them about the condition of hay fever which I had been subject to for years. The result of this was that they gave me a dose of Psorinum, which gave me relief from the pain in the jaw and a most intense and acute attack of hay fever which had lasted for three days, and I got back home at the close of the session and it gradually wore off.

Dr. Bell—You took enough.

Dr. Hitchcock—I took enough—I took one dose of Psorinum. Then my usual attack, which occurs about the 17th of August, did not occur until about five or six days later. When the hay fever came on I took a dose of Psorinum and it did not relieve me and I waited, prescribing for the symptoms as best I could, and finally, for an asthmatic attack at night I took a dose of Chamomile, which relieved me within a very few minutes. From that time during the rest of the seasom I was not affected by it. About four or five days ago I had a slight attack of hay fever, coming on with a very hot spell we had. I took nothing for it. It disappeared as the weather got colder. Whether I will have my usual attack of hay fever I do not know. I am not expecting it. I will look and see what effect it produced. What I wanted to call attention to was the alternation between the coryza and hay fever. I have a scaly eruption over the lower end of the sacrum.

Dr. Ballard—One day while riding down town I was introduced to a doctor by a druggist, and that druggist said, "I now want to see you doctors fight." There was no fight. This was in the spring. I did not meet the gentleman again until about August, and I found then he was in his blooming attack of hay fever and he challenged me to undertake it, and I said to him if he would come to the office I would do the best I could. He came in and I did the best I could, very much to his comfort, and the following season when his attacks reappeared I could not do anything with it. The symptoms were not pointed, and I prescribed for him at various times, but could not touch it at all. I asked him what he had been doing. Nothing. I put him under a good deal of cross-questioning. I learned that he had had that summer a reappearance of a foot-sweat which had not been noticed for a number of years, and he applied Iodoform and cured his foot-sweat and on came his hay fever. Then to go back into the history of this case, I learned that the hay fever was preceded by an asthma, and that asthma followed the suppression of this foot-sweat.

Dr. Bell—Was he an allopath?

Dr. Ballard—This is the gentleman who has just been talking to you (Dr. Hitchcock). He was an allopathic physician. Then we had a little talk and I told him all about it—how he had gone through with it. He disappeared from my view, went East, studied Homœopathy, and here he is.

Dr. Hitchcock—I want to say, Mr. President, that this salt rheum or eruption that I had on my hand was first suppressed with Iodoform. When I was a student in the college I applied the Iodoform ointment and suppressed it, but at that time with these symptoms I had also some slight attacks of hay fever, but nothing that was marked or strong did anything to improve my hay fever symptoms that occurred after the suppression of that eruption.

Dr. Ballard—I want to say, Mr. President, that I learned of the suppression. I want to say also that I gave him one dose of Silicea, and he had his asthma inside of forty-eight hours—a beautiful case of it.

The President—In consideration of the fact that Dr. Wells is about to leave us for the train, I want him to give us a few words of advice to keep us faithful for the next twelve months.

Dr. Wells—This is putting me in a good condition.

Dr. Brown—You always get through. I have never known you not to get through.

Dr. Wells—I wish I had been there all this time. I have been a good many years in this work, more than most of you who are now in it, I think. I have been all my life a learner, and only for a short period of my life I took up the office of a teacher. I am ready to receive advice from any one who is capable of giving it. I feel no call and no commission to advise my neighbors, but if I were to leave any word with this Association, it would be—so far as you have attained to the truth, abide by it. Stick to the truth, no matter what comes. Now I was quite refreshed but a moment ago when our brother Wesselhœft said psora. I do not know that there is a word that we are accustomed to use that has been so much laughed at as that word, but the idea that the word covers is a solid truth, whether we recognize it or not, and I would advise—it is hardly necessary because they will do it if I do not—that the great principles of the greatest master of healing that ever lived should be just those that we would take into our hearts, stick to them, defend them, and practice upon them, and live them, and we are sure in the end to live by and by a life which will be a life full of usefulness.

